# Impact of *KRAS* Mutations and Co-mutations on Clinical Outcomes in Pancreatic Ductal Adenocarcinoma

**DOI:** 10.21203/rs.3.rs-3195257/v1

**Published:** 2023-08-10

**Authors:** Abdelrahman Yousef, Mahmoud Yousef, Saikat Chowdhury, Kawther Abdilleh, Mark Knafl, Paul Edelkamp, Kristin Alfaro-Munoz, Ray Chacko, Jennifer Peterson, Brandon G. Smaglo, Robert A. Wolff, Shubham Pant, Michael S. Lee, Jason Willis, Michael Overman, Sudheer Doss, Lynn Matrisian, Mark W. Hurd, Rebecca Snyder, Matthew H.G. Katz, Huamin Wang, Anirban Maitra, John Paul Shen, Dan Zhao

**Affiliations:** 1 Department of Gastrointestinal Medical Oncology, The University of Texas MD Anderson Cancer Center, Houston, TX, USA; 2 Pancreatic Cancer Action Network, Manhattan Beach, Los Angeles, CA, USA; 3 Department of Genomic Medicine, The University of Texas MD Anderson Cancer Center, Houston, TX, USA; 4 Department of Data Engineering & Analytics, The University of Texas MD Anderson Cancer Center, Houston, TX, USA; 5 Sheikh Ahmed Center for Pancreatic Cancer Research, The University of Texas MD Anderson Cancer Center, Houston, TX, USA; 6 Department of Surgical Oncology, The University of Texas MD Anderson Cancer Center, Houston, TX, USA; 7 Department of Translational Molecular Pathology, The University of Texas MD Anderson Cancer Center, Houston, TX, USA

**Keywords:** *KRAS*, Mutation, Alleles, Subtypes, Co-mutations, Pancreatic cancer, Clinical outcome, OS, Survival, PDAC

## Abstract

The relevance of *KRAS* mutation alleles to clinical outcome remains inconclusive in pancreatic adenocarcinoma (PDAC). We conducted a retrospective study of 803 PDAC patients (42% with metastatic disease) at MD Anderson Cancer Center. Overall survival (OS) analysis demonstrated that *KRAS* mutation status and subtypes were prognostic (p<0.001). Relative to patients with *KRAS* wildtype tumors (median OS 38 months), patients with *KRAS*^*G12R*^ had a similar OS (median 34 months), while patients with *KRAS*^*Q61*^ and *KRAS*^*G12D*^ mutated tumors had shorter OS (median 20 months [HR: 1.9, 95% CI 1.2–3.0, p=0.006] and 22 months [HR: 1.7, 95% CI 1.3–2.3, p<0.001], respectively). There was enrichment of *KRAS*^*G12D*^ mutation in metastatic tumors (34% vs 24%, OR: 1.7, 95% CI 1.2–2.4, p=0.001) and enrichment of *KRAS*^*G12R*^ in well and moderately differentiated tumors (14% vs 9%, OR: 1.7, 95% CI 1.05–2.99, p=0.04). Similar findings were observed in the external validation cohort (PanCAN’s Know Your Tumor^®^ dataset, n=408).

## Introduction

Pancreatic ductal adenocarcinoma (PDAC) is projected to be the second leading cause of cancer death in US by 2040; with limited available treatment options for metastatic PDAC, the 5-year survival rate is less than 5%^1,2^. The median overall survival (OS) for the current standard of care chemotherapy (oxaliplatin, irinotecan, fluorouracil, and leucovorin [FOLFIRINOX]) is 11.1 months in the first line treatment of metastatic disease, with an objective response rate (ORR) of 31.6% and median progression-free survival (PFS) of 6.4 months^3,4^. The median OS for the other available first line chemotherapy regimen, gemcitabine/nab-paclitaxel, is 8.5 months with an ORR of 23% and median PFS of 5.5 months^5^. In the setting of second line treatment, the median OS with chemotherapy (liposomal irinotecan, fluorouracil and leucovorin) is only 6.1 months, with an ORR of 16% and median PFS of 3.1 months^6^. Better therapy for PDAC is urgently needed.

Among the identified genomic alterations (GAs) in PDAC, oncogenic *KRAS* mutations are the most common, occurring in close to 90% of patients, followed by *TP53*, *CDKN2A*, and *SMAD4*^7,8^. The majority of *KRAS* mutations are at codon 12, with the highest prevalence of G12D mutation (35%), followed by G12V (20–30%), G12R (10–20%), Q61H (~5%), 1%−2% G12C and other rare muations^9–12^. Targeting *KRAS* has been challenging for decades until the allosteric *KRAS*^G12C^ mutant-specific inhibition by covalent binding to the mutant cysteine beneath the switch-II region, which locks it in the inactive GDP bound form, has been discovered^13^. Exciting results from clinical trials of the *KRAS*^G12C^ inhibitors sotorasib (AMG510) and adagrasib (MRTX849) have been reported, and both have been approved by the US FDA for previously treated *KRAS*^G12C^-mutated advanced lung cancer. Moreover, efficacy of both sotorasib and adagrasib against PDAC has also been observed^14–18^. Sotorasib had a 21% ORR with a median PFS of 4.0 months in patients with pancreatic cancer who had received chemotherapy previously^19^. Adagrasib monotherapy had an ORR of 33.3% with a median PFS of 5.4 months (95% CI 3.9–8.2) and a median OS of 8.0 months ( 95% CI 5.2–11.8) in pancreatic cancer patients refractory to chemotherapy (n=21)^20^. More excitingly, preclinical development of a *KRAS*^G12D^ inhibitor (MRTX 1133) has shown promising results and MRTX 1133 is currently in phase 1 clinical trial^21^. Pan *KRAS* inhibitor RMC-6236, which binds to the chaperone protein, cyclophilin A, and active GTP-bound RAS (RAS ON inhibitor) are also being tested in patients with *KRAS*^G12^ mutations, including G12D, G12V, G12R, G12A, or G12S mutations (NCT05379985). Moreover, T cell therapy with *KRAS*^G12D^-targeting T cell receptors (TCRs) caused tumor regression in a pancreatic cancer patient, and T cells with TCRs targeting other *KRAS* mutations, such *KRAS*^G12V^, are under development ^22,23^. We are at a breakthrough point in attempts to target *KRAS* in pancreatic cancer. The remaining challenges include the short duration of response and primary/secondary resistance to *KRAS* inhibition. Additionally, while multiple genomic and non-genomic factors have been associated with resistance to *KRAS* inhibitors, such as co-mutations of *KEAP1/STK11* with *KRAS* as observed in patients with lung cancer, comutations *KEAP1/STK11* mutations are rare in pancreatic cancer; little is known about the landscape of *KRAS* mutations and co-mutations in pancreatic cancer or their impact on clinical outcomes^12,24,25^.

*KRAS*-mutated cancers are heterogeneous with different mutation allele subtypes and co-mutations^26–28^. Each *KRAS* mutation allele subtype has unique biochemical and clinicopathological features, and the differences between the mutation subtypes and commutations in pancreatic cancer have not been well studied^26–29^. The *KRAS*^*G12D*^ mutation has an intrinsic wildtype and SOS1 guanine exchange activities while the *KRAS*^*Q61*^ mutation has deficiencies in GTP hydrolysis^27,30^. The *KRAS*^*G12R*^ mutation, which accounts for approximately 15% in pancreatic cancer but less than 1% in lung cancer, was reported to be associated with different downstream signaling pathways from other *KRAS* mutations^27^. The *KRAS*^*G12D*^ mutation was reported to be more immune suppressive with shorter survival in lung cancer and pancreatic cancer^31,32^. Moreover, it has been reported that the mutations that co-mutations with *KRAS* vary with the *KRAS* mutation alleles in patients with lung cancer, and these different patterns of co-mutation with *KRAS* differentially affect clinical outcomes^33^. For example, co-mutation of *KEAP1/STK11* was more common in patients with *KRAS*^*G13*^-mutated lung cancer than *KRAS*^*G12D*^-mutated lung cancer, and the co-mutation of *KEAP1/STK11* with *KRAS*^*G13*^ was associated with poor prognosis and treatment resistance^28^.

Research to date on the impact of *KRAS* allele subtypes and co-mutations on clinical outcome in PDAC has been limited, and the conclusions remain controversial. *KRAS*^*G12D*^ was reported to be associated with worse OS compared that among with patients with *KRAS*^*G12R*^-mutated pancreatic cancer in a single institutional study (n=126); however, within the *KRAS*^*G12R*^-mutated PDAC group, those with co-occurring *PI3K* pathway mutations experienced worse OS^34^. Meanwhile, there was no statistically significant difference in OS between different *KRAS* mutation alleles in another study^12^. Our institution has collaborated with the data science firm Syntropy to deploy the Palantir Foundry software platform for extraction and analysis of merged clinical data and laboratory data across a variety of platforms, including the Electronic Health Record (EHR); molecular testing/next generation sequencing (NGS), pathology, and radiology results; and tumor registry data^35–37^. Together with the development of data science tools such as natural language processing (NLP) and the increased use of NGS in pancreatic cancer, the Foundry platform now gives us the ability to analyze large datasets comprising real-world clinical and molecular information to dissect the heterogeneity of *KRAS*-mutated pancreatic cancer. In this study, we illustrate the co-mutation landscape of *KRAS* mutations and the allele specific differences of *KRAS*-mutated pancreatic cancer with clinical outcome in our institution. In addition, we validated our findings in an external cohort from the Pancreatic Cancer Action Network (PanCAN)’s Know Your Tumor^®^ (KYT) Dataset^38^.

## Results

### Patient Characteristics

**Figure 1.**
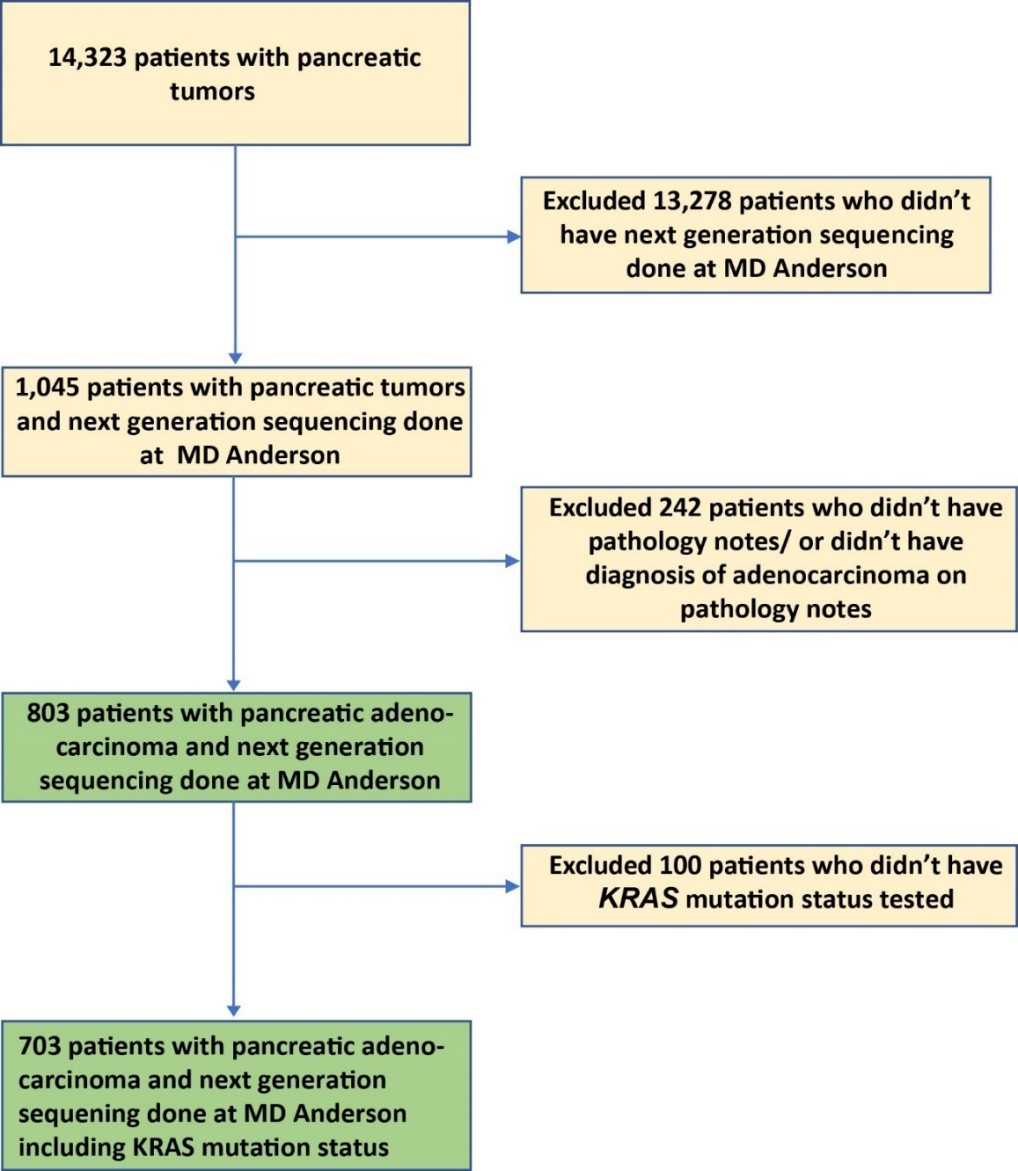
Flowchart diagram showing cohort patients selection. Abbreviations include MD Anderson (MD Anderson Cancer Center)

A total of 803 patients with PDAC who had tumor tissue somatic mutation testing at MD Anderson were identified (Fig 1); the demographic and clinical characteristics of this cohort are summarized in Table 1. The median age was 63 years (range 26–86), 43% were female, and 29.3% had a smoking history (current or former). A total of 336 (42%) patients had documented stage IV disease at the time of their initial diagnosis, and 321 (40%) had poorly differentiated tumors. *KRAS* gene mutation status was tested in 703 patients, including 302 with stage IV disease; 578 (82%) were positive for mutated *KRAS*. In addition to *KRAS, TP53* was tested in 604 patients, 418 (69%) of whom were positive; *TP52*. *CDKN2A* was tested in 509 patients, 102 (20%) of whom were positive; and *SMAD4* was tested in 536 patients, 68 (13%) of whom were positive. The median follow-up time from initial diagnosis was 41 months. Median OS of the entire cohort of 803 patients at MD Anderson was 19 months (range 0.07–348).

### *KRAS* Mutation Status and Allele Subtype Association with OS

**Figure 2.**
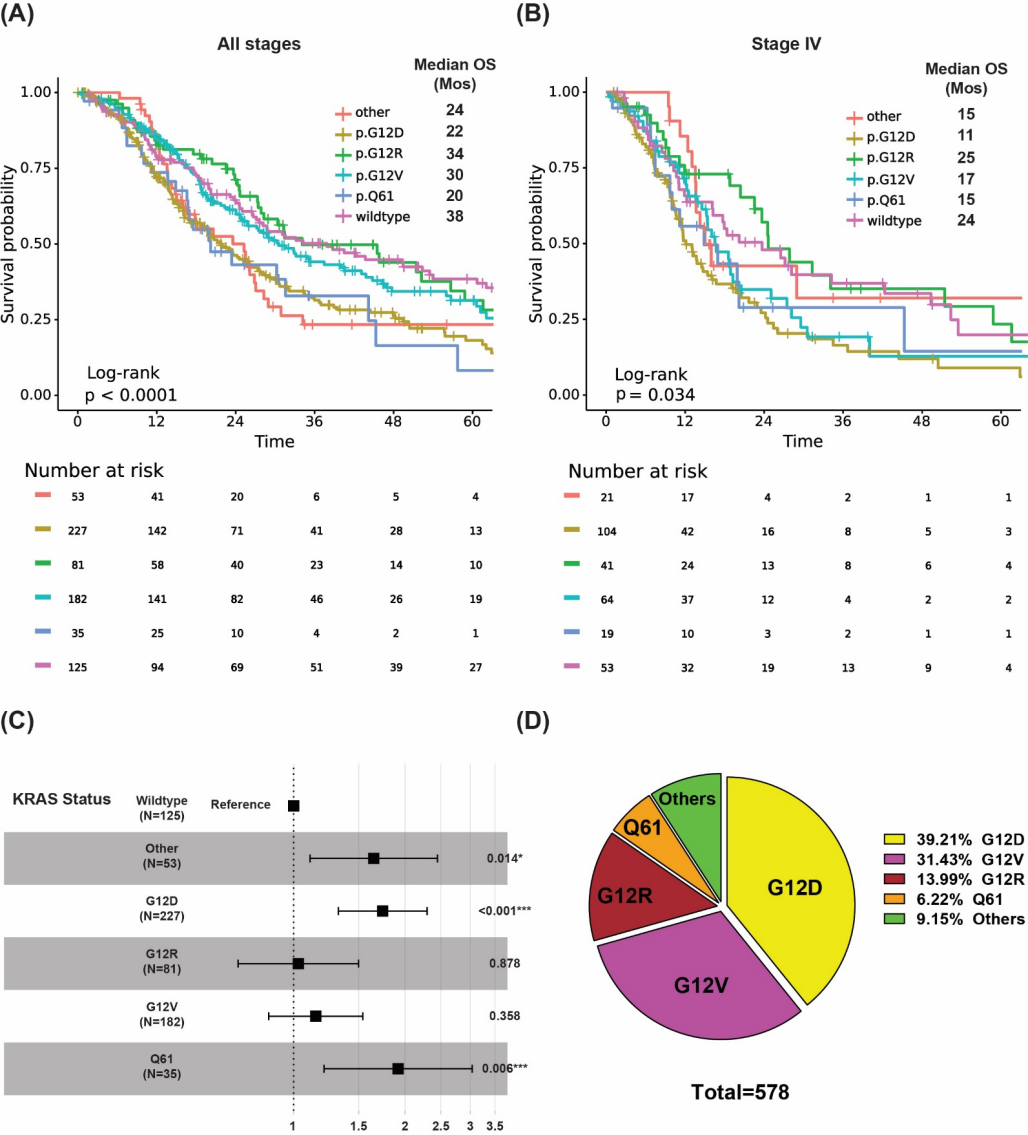
Overall survival (OS) with *KRAS* mutations and mutation subtypes. **(A)** KM OS curves of all patients, and stage IV patients only **(B)** with *KRAS*-mutated PDAC **(C)** Univariate analysis of OS with *KRAS* mutation subtypes and **(D)** Frequencies of different *KRAS* mutations in patients with *KRAS*-mutant PDAC (n=578)

Among the 578 whose tumors tested positive for a *KRAS* mutation, 227 had *KRAS*^G12D^ (39%), 182 had *KRAS*^G12V^ (31%), 81 had *KRAS*^G12R^ (14%), 35 had *KRAS*^Q61^ (6%), and 53 had other uncommon *KRAS* variants (9%) (Fig 2D). The Kaplan-Meier (KM) analysis of OS in all 703 patients with known *KRAS* mutation status, with all stages include, demonstrated that *KRAS* mutation status and subtype was prognostic of OS (p<0.001) (Fig 2A); patients with *KRAS* wildtype tumors had a median OS of 38 months (reference), patients with *KRAS*^G12R^ tumors had a median OS of 34 months (HR: 1, 95% CI 0.71–1.5, p=0.88), patients with *KRAS*^Q61^ tumors had a median OS of 20 months (HR: 1.9, 95% CI 1.2–3.0, p=0.006), and patients with *KRAS*^G12D^ tumors had a median OS of 22 months (HR: 1.7, 95% CI 1.3–2.3, p<0.001) (Fig 2C). When limited to patients with stage IV metastatic disease (n=302), *KRAS* mutation remained significantly associated with OS (p=0.034) (Fig 2B). Again, patients with *KRAS*^Q61^ and *KRAS*^G12D^ mutations had shorter median OS, with 15 and 11 months respectively, relative to those with *KRAS*^G12R^ mutations and *KRAS* wildtype tumors (median OS of 25 and 24 months respectively). *KRAS*^G12D^ (HR= 1.7, 95% CI 1.1–2.6, p=0.009) was associated with significantly worse OS relative to *KRAS* wildtype tumors.

### *KRAS* Mutation Allele Subtype Association with Stage and Tumor Differentiation

**Figure 3.**
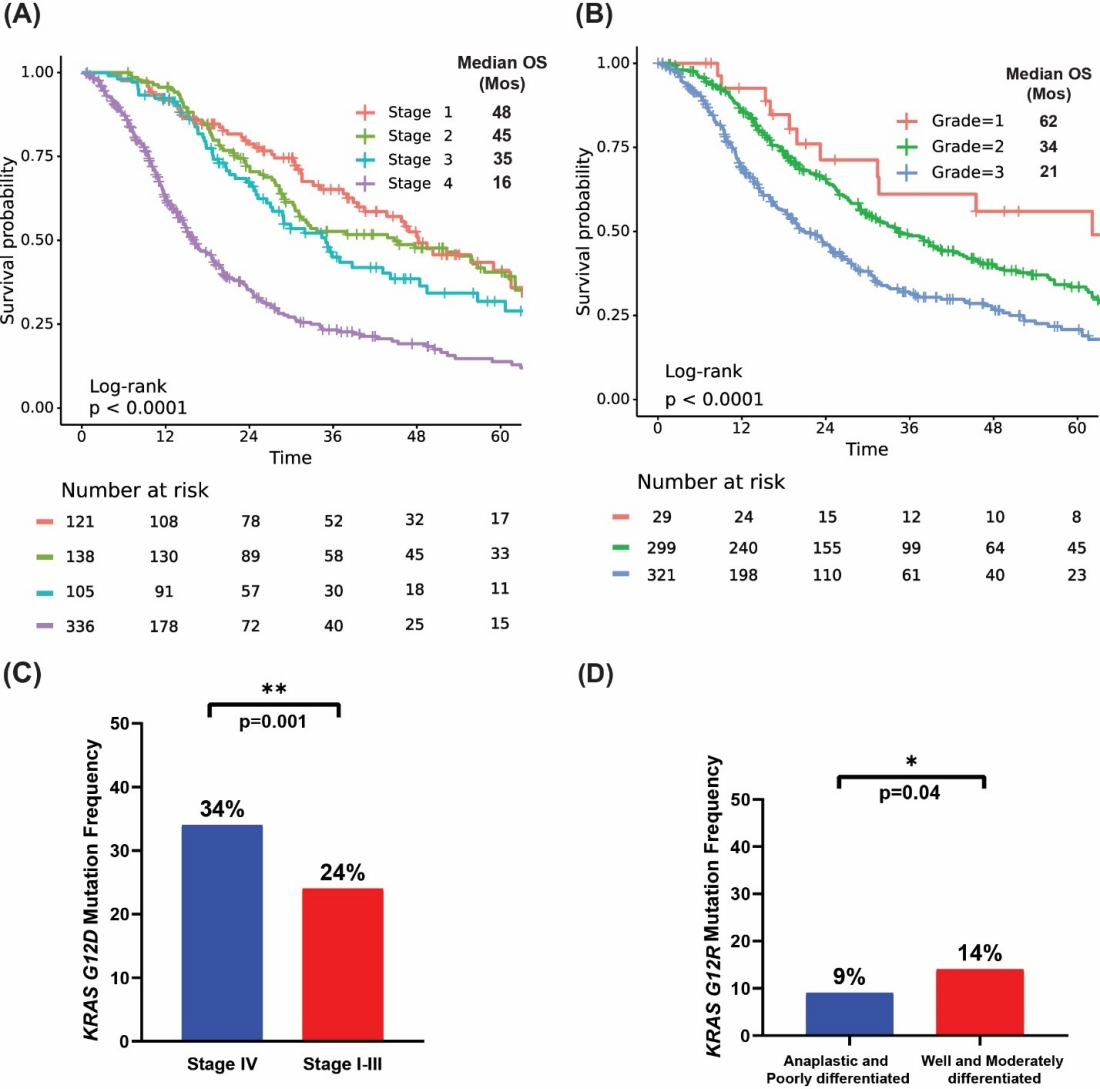
**(A)** KM OS curves for tumor stage of our cohort. **(B)** KM OS curves for tumor histopathological grade of our cohort. **(C)** Bar plot showing enrichment of *KRAS*^G12D^ mutation in metastatic disease. **(D)** Bar plot showing enrichment of *KRAS*^*G12R*^ in well and moderately differentiated tumors.

Advanced disease stage was associated with decreased OS (p<0.001). Patients with stage IV PDAC had median OS of 16 months (HR: 3.3, 95% CI 2.4–4.4) while patients with stage I PDAC had a median OS of 48 months (Fig 3A). In the full cohort of patients with known *KRAS* mutation status (all stages included), tumor histopathology was also prognostic of OS (p<0.001); poorly differentiated/anaplastic tumors had shorter overall survival (median OS = 21 months; HR: 2.3, 95% CI 1.4–3.9) than patients with well-differentiated tumors (median OS = 62 months) (Fig 3B). We also found a greater prevalence of *KRAS*^*G12D*^ mutations in patients with metastatic disease (stage IV) vs patients with localized disease (stage I-III) (34% vs. 24%, OR:1.7, 95%CI:1.2–2.4, p=0.001) (Fig 3C), as well as an increased prevalence of *KRAS*^*G12R*^ mutations in well and moderately differentiated tumors vs poorly differentiated/anaplastic tumors (14% vs. 9%, OR:1.7, 95% CI: 1.05–2.99, p=0.04) (Fig 3D).

### *KRAS* Co-mutations and OS

**Figure 4.**
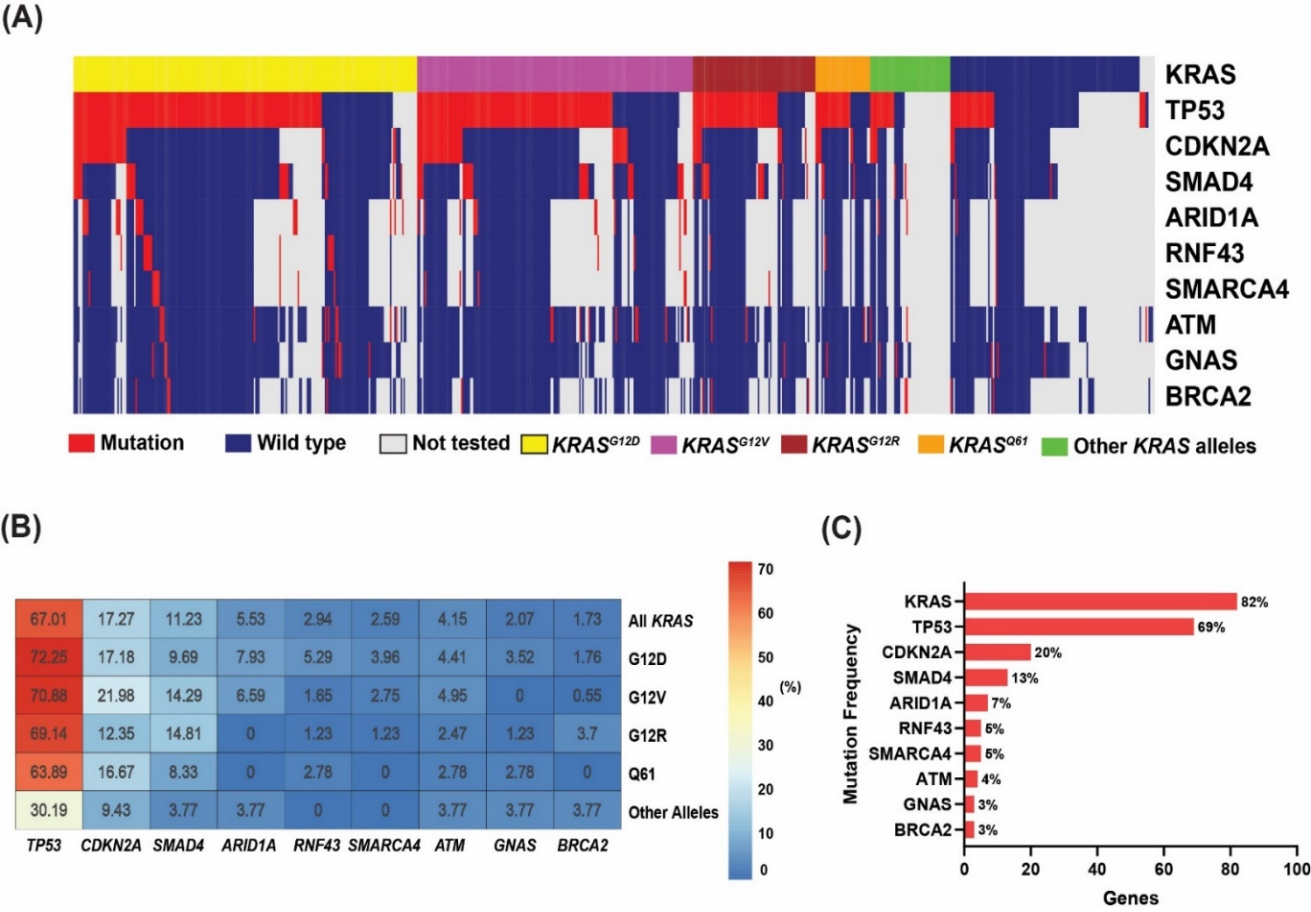
**(A)** Oncoplot showing the distribution of different *KRAS* mutational subtypes with the different genes in our cohort. **(B)** Heatmap showing the co-mutation landscape of the different *KRAS* mutation subtypes with the different genes and their frequencies. **(C)** Bar plot showing the most frequently mutated genes in our cohort.

**Figure 5.**
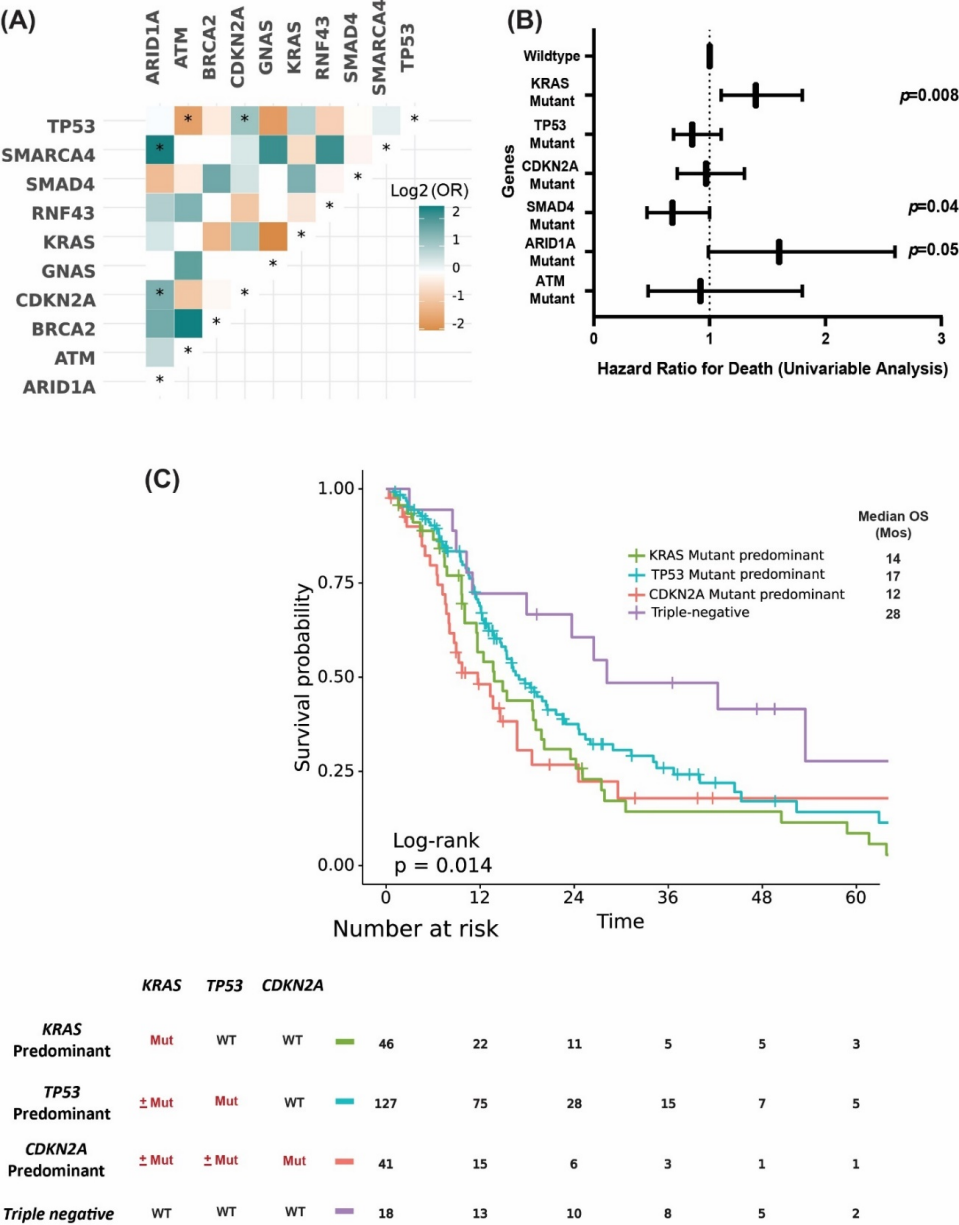
**(A)** Co-mutation analysis of the MD Anderson cohort. Associations between prevalent driver mutations were assessed using the Fisher’s exact method and a significant FDR-corrected p indicated by asterixis (*FDR-corrected p <0.1). **(B)** Forest plot showing HR for death (from a univariable analysis) for driver mutations in our cohort, wildtype of each gene was used as reference. **(C)** KM OS analysis in patients with metastatic PDAC stratified by molecular subtype.

The top detected GAs (Fig 4A) were sorted by the detected positive rate of GAs among tested patients (the number of tested patients for each gene varied due to different gene panels in the testing platforms). *KRAS* (82%, N=578 of 703), *TP53* (69%, N=418 of 608), *CDKN2A* (20%, N=102 of 509), *SMAD4* (13%, N=68 of 536), and *ARID1A* (7%, N=34 of 482) were the most commonly mutated genes in the MD Anderson cohort (Fig 4C). *TP53* was the most frequently detected co-mutation with *KRAS*, with a 67% co-positive rate for *TP53*, followed by *CDKN2A* (17%), *SMAD4* (11%), and *ARID1A* (6%) (Fig 4B). In the co-mutation analysis, *KRAS* was found to be frequently co-mutated with *TP53* (OR: 1.77, 95% CI 0.85–3.6, false discovery rate (FDR)-corrected p=0.29), and *CDKN2A* (OR: 2.05, 95% CI 0.71–8.13, FDR-corrected p=0.47). Interestingly, *KRAS* and *GNAS* were mutually exclusive (OR: 0.23, 95% CI 0.07–1.05, FDR-corrected p=0.14) while *TP53* and *ATM* were mutually exclusive (OR: 0.31, 95% CI 0.12–0.81, FDR-corrected p=0.095). (Fig 5A). Moreover, *TP53* and *CDKN2A* were frequently co-occurring (OR: 2.11, 95% CI 1.17–4.04, FDR-corrected p=0.095). Also, *ARID1A* was found to be significantly co-mutated with *CDKN2A* (OR: 2.7, 95% CI 1.18–6.02, FDR-corrected p=0.095), and with *SMARCA4* (OR: 5.17, 95% CI 1.15–18.44, FDR-corrected p=0.1).

In univariate Cox proportional hazards analysis for the most commonly mutated genes in the MD Anderson cohort, *ARID1A* mutation was associated with poor OS, with median OS of 18 months in patients with *ARID1A*-mutated tumors vs 31 months in patients with *ARID1A* wildtype tumors (HR: 1.6, 95% CI 0.99–2.6, p=0.05). However, *SMAD4* mutant tumors had better OS than *SMAD4* wildtype tumors (median OS 35 and 27 months, respectively, HR: 0.67, 95% CI 0.46–0.99, p= 0.046) (Fig 5B). Interestingly, while none of the patients with *KRAS*^*G12R*^-mutated tumors had *ARID1A* co-mutation, *ARID1A* co-mutation was observed in 8% of *KRAS*^*G12D*^ mutated tumors (p=0.02). Conversely, *SMAD4* co-mutation was observed in 15% of the patients with *KRAS*^*G12R*^-mutated tumors compared with 10% in in patients with *KRAS*^*G12D*^-mutated tumors (p=0.22) (Fig 4B).

In patients with metastatic disease and known *KRAS*, *TP53*, and *CDKN2A* mutation status (n=232), we classified four distinct molecular subtypes of metastatic PDAC (Fig 5C): (1) *KRAS* mutant predominant (*KRAS* mutant, *TP53* wildtype*/CDKN2A* wildtype) (n=46/232), (2) *TP53* mutant predominant (*TP53* mutant, *KRAS* mutant or wildtype/*CDKN2A* wildtype) (n=127/232), (3) *CDKN2A* mutant predominant (*CDKN2A* mutant, *KRAS* mutant or wildtype/*TP53* mutant or wildtype) (n=41/232), and (4) triple negative (all *KRAS, TP53*, and *CDKN2A* wildtype) (n=18/232). Patients with triple negative (*KRAS-/TP53-/CDKN2A-)* tumors had the longest median OS (28 months), while *CDKN2A* predominant group had worst OS (median 12 months) with *TP53* predominant group (median 17 months) and *KRAS* predominant group (median 14 months) had intermediate OS (p=0.014) (Fig 5C).

### PanCAN’s Know Your Tumor^®^ Dataset

**Figure 6.**
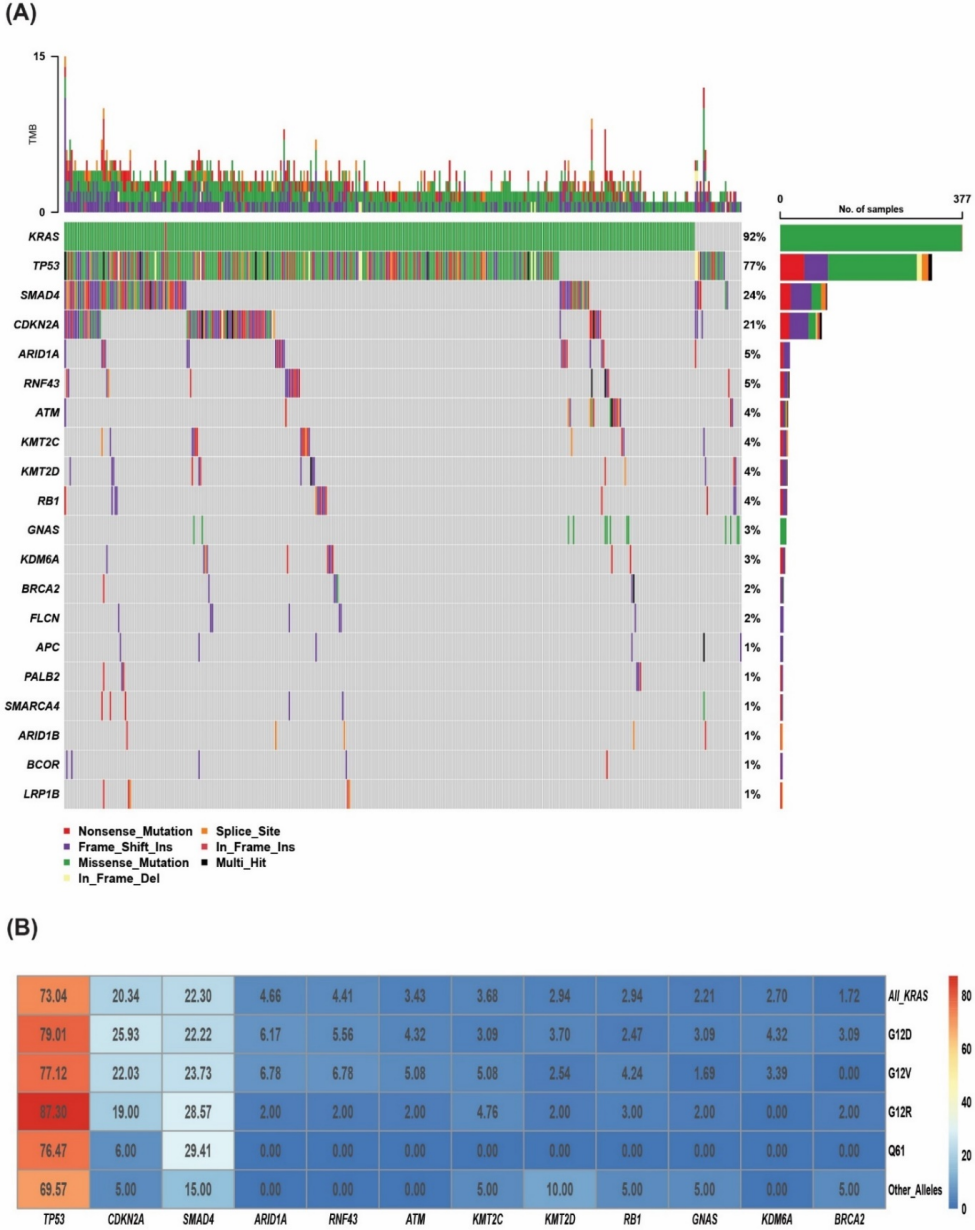
**(A)** Oncoplot showing the somatic mutation distribution across the KYT cohort. **(B)** Heatmap showing the co-mutation landscape of the different *KRAS* mutation subtypes with the different genes and their frequencies in KYT cohort.

To validate our findings, an external cohort from PanCAN’s KYT dataset (n=408) was analyzed. Baseline characteristics of patients in the KYT cohort are summarized in Table 2. The median age at the time of diagnosis was 65 years (range 36–88). 46% were female and 54% were male. The median follow-up time from diagnosis was 15 months. Disease staging information was not available in majority of the patients in this cohort (59.8%). 23.8% (n=97) patients had documented stage IV disease at the time of diagnosis. Median overall survival in all the patients was 22 months (range 0.2–93 months). *KRAS* (92%), *TP53* (77%), *SMAD4* (24%), *CDKN2A* (21%), and *ARID1A* (5%) were the most commonly mutated genes in the PanCAN cohort (Fig 6A).

**Figure 7.**
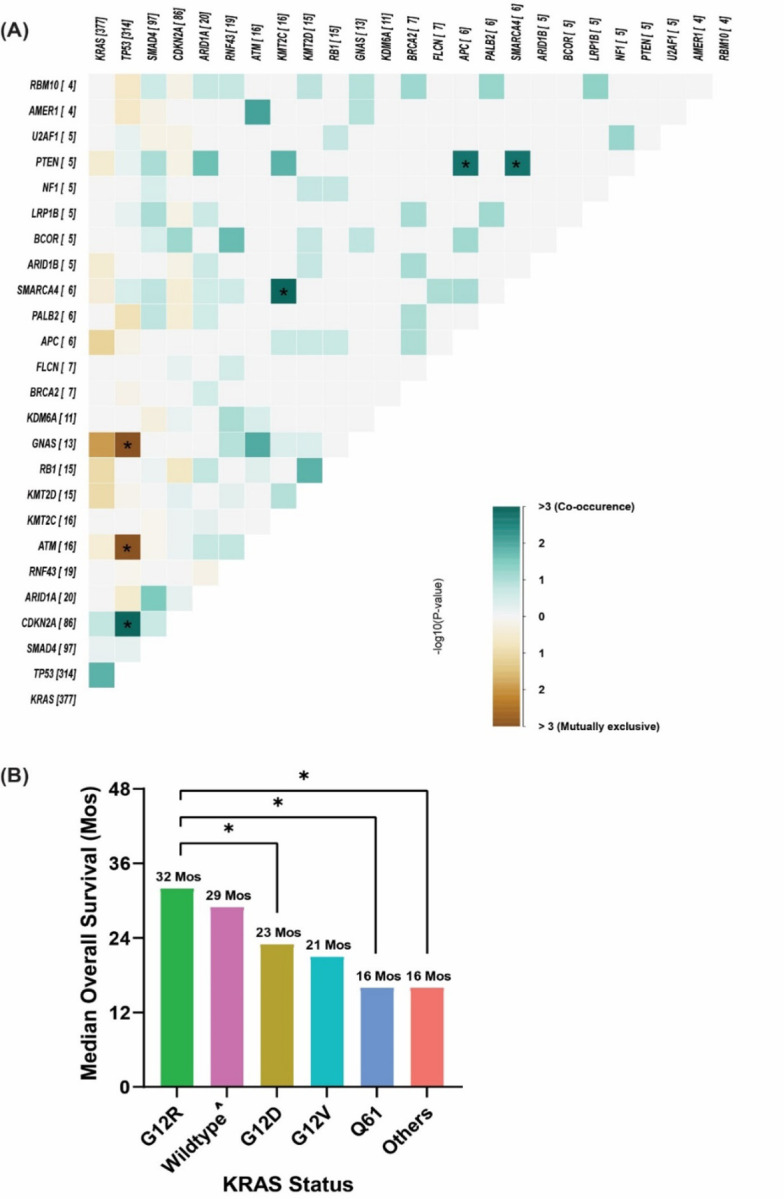
**(A)** Co-mutation analysis of KYT cohort, associations between prevalent driver mutations were assessed using the Fisher’s exact method and a significant FDR-corrected p indicated by asterixis (*FDR-corrected p <0.1). **(B)** Bar plot showing the difference in median overall survival between different *KRAS* mutation subtypes. (*) indicate p<0.05 using log-rank test for survival. **(^)** Wildtype: indicate no pathogenic mutations were detected.

Similar to the MD Anderson cohort, *TP53* was the most frequently detected co-mutation with *KRAS*, (73% positive rate), followed by *CDKN2A* (20%), *SMAD4* (22%), and *ARID1A* (5%) (Fig 6B). In the co-mutation analysis (Fig 7A), *KRAS* was found to be frequently co-mutated with *TP53* (OR: 2.6, FDR-corrected p= 0.18), and *CDKN2A* (OR: 2.6, FDR-corrected p= 0.84). *TP53* and *CDKN2A* were frequently co-occurring (OR: 3.54, FDR-corrected p=0.009). *TP53* was mutually exclusive with both *ATM* (OR: 0.04, FDR-corrected p= 9.8E-07) and *GNAS* (OR: 0.05, FDR-corrected p= 6.65E-05). *KRAS* and *GNAS* were also mutually exclusive (OR: 0.17, FDR-corrected p= 0.18) (Fig 7A). *KRAS*^G12R^ was associated with significantly longer median OS (32 months) than *KRAS*^Q61^ (16 months, HR: 2.6, 95% CI 0.88–7.8, p=0.02) and *KRAS*^G12D^ (23 months, HR: 1.68, 95% CI 1.06–2.65, p=0.04) (Fig 7B).

## Discussion

In this study, we analyzed the impact of *KRAS* mutation status, *KRAS* allele subtypes, and co-occurring mutations on clinical outcome of patients with PDAC in two real-world datasets. The study included 803 patients who had been tested for somatic tumor mutations at MD Anderson Cancer Center and an external cohort (n=408 of patients with pancreatic cancer from the PanCAN KYT^®^ dataset. We found that *KRAS* mutation status and allele subtypes were associated with OS; median OS was longer in patients with *KRAS* wildtype and *KRAS*^*G12R*^-mutated tumors compared to median OS in patients with *KRAS*^*G12D*^ or *KRAS*^*Q61*^-mutated tumors. We illustrated the co-mutation landscape with *KRAS* mutation. We also found that *ARID1A* mutation was associated with worse OS and *SMAD4* was associated with better OS. We found *TP53* and *ATM* mutation were mutually exclusive. There was a higher rate of *ARID1A* mutation in *KRAS*^*G12D*^ compared with *KRAS*^*G12R*^ patients. We also found enrichment of *KRAS*^*G12D*^ in metastatic disease and enrichment of *KRAS*^*G12R*^ in well to moderately differentiated tumors.

Among the 803 patients with PDAC analyzed, 703 were tested for *KRAS* mutation at MD Anderson (Fig 1). The overall positive rate for *KRAS* mutation was 82% (n=578) with the most common mutation of *KRAS*^G12D^ (39%), followed by *KRAS*^G12V^ (31%), *KRAS*^G12R^ (14%), *KRAS*^Q61^ (6%), and other uncommon *KRAS* variants (9%) (Fig 2D). There were differences in OS with *KRAS* mutation status and allele subtypes in the overall population (Fig 2A) and stage IV patients who had tested for *KRAS* (n=302) (Fig 2B). Compared with wildtype patients regardless of disease stages, *KRAS*^*G12D*^ (median OS 22 months, HR: 1.7, 95% CI 1.3–2.3, p<0.001) and *KRAS*^*Q61*^ (median OS 20 months, HR: 1.9, 95% CI 1.2–3.0, p=0.006) had worse survival. *KRAS*^*G12R*^ mutated patients (median OS 34 months, HR: 1, 95% CI 0.71–1.5, p=0.88) had similar OS as wildtype patients (median OS 38 months, reference) (Fig 2C). The external cohort of PanCAN KYT^®^ dataset (n=408) validated that *KRAS*^G12R^ was associated with best median OS (32 months), while *KRAS*^Q61^ (16 months, HR: 2.6, 95% CI 0.88–7.8, p=0.02) and *KRAS*^G12D^ (23 months, HR: 1.68, 95% CI 1.06–2.65, p=0.04) were associated with shorter median OS (Fig 7B). Our results were consistent with the previous report of significantly longer OS (HR 0.55) in patients with *KRAS*^*G12R*^-mutated PDAC (n=23) compared with those with non- *KRAS*^*G12R*^ PDAC (n=88)^34^. Another study comparing *KRAS*^*G12C*^ (n=30) and other *KRAS* mutations reported longer median OS (p=0.03) for *KRAS* wildtype patients (n=91) which was consistent with our findings of better survival in *KRAS* wildtype patients^12^. The previously reported study analyzed the OS of metastatic PDAC from starting the first line therapy and did not show statistically significant difference between other *KRAS* alleles while compared against *KRAS*^*G12C*^ patients^12^. Due to the low frequency of *KRAS*^*G12C*^ mutation, we grouped the *KRAS*^*G12C*^ with other uncommon mutations. In our cohort, OS was defined from initial diagnosis and there was enrichment of *KRAS*^*G12D*^ mutation in metastatic disease (stage IV) (OR: 1.7, 95% CI 1.2–2.4, p=0.001) (Fig 3C). Our data suggested worse outcome of *KRAS*^G12D^ tumors. This is consistent with the study of 356 resected patients with PDAC, which reported that *KRAS* mutation had worse disease-free survival (DFS) (median 12.3 months) and OS (median 20.3 months) compared with wildtype (DFS 16.2 months and OS 38.6 months) and poor outcome in *KRAS*^*G12D*^ patients (median OS 15.3 months)^39^. The mechanisms of why *KRAS*^*G12D*^ had worse prognosis is not fully understood beyond the comutations. More immunosuppressive tumor microenvironment (TME) with *KRAS*^*G12D*^ tumors was found in lung cancer^28,31^. In *KRAS*^*G12D*^ mutation driven PDAC mice model, there were immune suppressive cytokines IL-4 and IL-13 and remodeling of myeloid cell composition in TME^40,41^. In PDAC mice models treated with the *KRAS*^G12D^ inhibitor MRTX1133, increased macrophages (CD11b and F4/80+) in the TME with decreased the total myeloid cells was observed^42^. Correlative tissue and blood samples for potential *KRAS* mutation allele specific immune features were not included in this project and could be a future direction in patients with PDAC.

*KRAS*^*G12R*^ existed most commonly in PDAC (~15%) with low frequency in other cancer types^12^. It has distinct biochemical features from *KRAS*^*G12D/V*^ with altered switch-II structure that could not activate p110α/PI3K directly^43^. We found the median OS of *KRAS*^*G12R*^ mutated patients was comparable to wildtype patients and longer than *KRAS*^*G12D*^ or *KRAS*^*Q61*^ mutated patients. There was enrichment of *KRAS*^*G12R*^ mutation in well and moderately differentiated tumors vs poorly differentiated/anaplastic tumors (OR: 1.7, 95% CI 1.05–2.99, p=0.04) (Fig 3D), which suggested less aggressive biology and better outcome for the *KRAS*^*G12R*^-mutated tumors. On the other hand, *KRAS*^*Q61*^ mutants had decreased GTP hydrolysis rate with high RAF-dependent MEK phosphorylation and it did not response to SOS1 inhibition^29,44^. While *KRAS*^*Q61*^ mutants had shorter median OS in our cohort, little is known about the clinical features of *KRAS*^*Q61*^ mutants. To our best knowledge, this is the first study to report worse OS with *KRAS*^*Q61*^ which could be consistent with its biochemical features. Due to the rarity of *KRAS*^*Q61*^ mutations, we grouped different *KRAS*^*Q61*^ mutations together, which may be mutant specific^45^. The clinical and molecular features of *KRAS*^*G12R*^ and *KRAS*^*Q61*^ mutated PDAC warrant further and larger studies which could help the development of *KRAS* allele specific inhibitors such as the *KRAS*^*G12R*^ inhibitor^46^.

Co-mutations with *KRAS* could be one of the contributing factors for the allele specific clinical outcome in PDAC. *KEAP1* co-mutation with *KRAS* in lung cancer was associated with early progression on *KRAS*^*G12C*^ inhibitor sotorasib^25^. Co-occurrence of other mutations in PDAC were common and the disease progression model was proposed with early *KRAS* mutation followed by *CDKN2A* then loss of *TP53* and *SMAD4*^47,48^. Our data was consistent with previous reports that *TP53* (67%) was the most common co-mutation with *KRAS* followed by *CDKN2A* (17%), *SMAD4* (11%), and *ARID1A* (6%) (Fig 4B)^12^. We tested the *KRAS/CDKN2A/TP53* disease progression model by classified four distinct molecular subtypes of metastatic patients in patients who had tested for *KRAS*, *TP53*, and *CDKN2A* (n=232). We found patients with triple negative (*KRAS-/TP53-/CDKN2A-)* tumors demonstrated better OS (median 28 months) with *CDKN2A* predominant type had the worst OS (median OS12 months, p=0.014) (Fig 5C). In our study, *CDKN2A* mutation included any mutation either missense or deletion of *CDKN2A*. Germline *CDKN2A* mutation increased the risks of melanoma and pancreatic cancer and somatic *CDKN2A* loss was common in pancreatic cancer^49–51^. *CDKN2A* loss had worse survival (median DFS 11.5 and OS 19.7) in patients with resected PDAC compared with wildtype patients (median DFS 14.8 and median OS 24.6)^39^. In another study of 100 patients with both metastatic and nonmetastatic PDAC, *CDKN2A* mutations were also associated with shorter OS (22 months vs 35 months; P = 0.018)^52^. In *KRAS*-mutated lung cancer, *CDKN2A* mutation was associated with worse survival on imunotherapy^53^. In mice model, *CDKN2A* loss accelerated *KRAS*^*G12D*^ driven tumor^54^. Targeting *CDKN2A* in *KRAS*-mutated PDAC is under investigation yet clinical activities of CDK4/6 inhibitors in early phase trials was not seen^55,56^. The location of the methylthioadenosine phosphorylase gene (*MTAP*) is adjacent to *CDKN2A* and majority of PDAC with *CDKN2A* loss also had *MTAP* loss^57–59^. The surrogate role of *CDKN2A* is not clear with low reported rate of *MTAP* loss in our cohort, due to the detection method for *MTAP* loss is not currently validated by comparative genomic hybridization for pancreatic cancer in our NGS testing panel.

Univariate OS analysis in our study did not show statistically significant association of co-mutation with *TP53* or *CDKN2A* mutation with OS but revealed that *ARID1A* mutant was associated with poor OS (median 18 vs 31 months, HR: 1.6, 95% CI 0.99–2.6, p=0.05) and *SMAD4* mutant was associated with better OS (median 35 vs 27 months, HR: 0.67, 95% CI 0.46–0.99, p=0.046) (Fig 5B). *SMAD4* is a tumor suppressor gene and inconsistent results were reported about the prognostic value of *SMAD4*^39,60–62^. *SMAD4* inactivation in resected PDAC was associated with poor prognosis while the meta-analysis did not show its association with OS^61,62^. Our data showed 13% *SMAD4* mutation rate and it was associated with better OS. Further studies with larger sample size and different populations are needed to understand the different results. *ARID1A* was found to be significantly co-mutated with *CDKN2A* (OR: 2.7, 95% CI 1.18–6.02, FDR-corrected p=0.095), and with *SMARCA4* (OR: 5.17, 95% CI 1.15–18.44, FDR-corrected p=0.1). *KRAS*^*G12R*^ mutated patients had lower *ARID1A* compared with *KRAS*^*G12D*^ (0% vs 8% in p=0.02) (Fig 4B). Similar findings were also observed in the validation cohort of the PanCAN KYT^®^ dataset. Both *ARID1A* and *SMARCA4* are Switch/Sucrose Nonfermentable (SWI/SNF) chromatin remodeling complex genes which are important in epigenetic reprogramming in PDAC^63^. Context specific tumor suppressive or oncogenic function of SWI/SNF chromatin regulation was noticed in PDAC^64,65^. In mice models, disrupted *ARID1A* promoted the carcinogenesis from *KRAS*-mutated premalignant intraductal papillary mucinous neoplasms (IPMN) to PDAC^44^. In *KRAS-*mutated colon cancer, a similar tumor supporting role of *ARID1A* was required for MEK/ERK signaling^66^. Our results of worse OS with *ARID1A* support the oncogenic role of *ARID1A* and targeting *ARID1A* in PDAC. ARID1A regulates DNA damage checkpoints and sensitizes cells to DNA damage response (DDR) targeting agents^67–69^. ATM-TP53 signaling pathway is critical in DDR targeting in pancreatic cancer^70^. Interestingly, in both of our cohorts, *TP53* mutation was mutually exclusive with *ATM* mutation. *TP53* and *ATM* mutation exclusivity was reported in mantle cell lymphoma with distinct clinical impact and sensitive to the protein arginine methyltransferase 5 (PRMT5) inhibitor^71,72^. Our findings of worse OS with *ARID1A* mutation and mutual exclusivity of *TP53* and *ATM* mutation in PDAC provided insights on therapeutic vulnerabilities of PDAC.

In summary, we reported the *KRAS* mutation allele specific clinical outcome in PDAC using a single institution retrospective study and an external validate cohort. Our findings suggested that *KRAS* targeting, and combination strategies may warrant the mutant allele specific approaches with consideration of the co-occurring mutations with *KRAS*.

## Limitations

The limitations of this study are heterogeneities in both patient populations and tumor mutation testing methods and gene panels. Only patients who had tissue molecular testing done at MD Anderson were included in this study while patients who had tested by other panels were not included. It is a retrospective study in a single tertiary cancer institution with ascertainment bias. The external validation cohort had limited clinical information. Treatment information was not available. Tumor genomic factors may not be the main contributor for *KRAS* mutation allele specificities. Correlative tissue and blood samples from patients for other non-genomic factors accounting for the differences in clinical outcome are not included in this study.

## Conclusion

In our analysis of 803 patients with PDAC, we found that *KRAS* mutation status and mutation allele subtypes were associated with OS. Patients with *KRAS* wildtype and *KRAS*^*G12R*^-mutated tumors survived longer than patients with *KRAS*^*G12D*^ or *KRAS*^*Q61*^-mutated tumors, and this observation was confirmed in an external validation cohort. We also found enrichment of *KRAS*^*G12D*^ mutations in patients with metastatic disease and *KRAS*^*G12R*^ mutations in patients with well to moderately differentiated tumors. We found co-mutations could contribute to the *KRAS* allele specific outcome. We found worse OS in *ARID1A* mutated patients and lower co-mutation rate of *ARID1A* in *KRAS*^*G12R*^. Our findings of different clinical outcomes by *KRAS* mutation subtypes and co-mutations status suggest allele- and co-mutation-specific impact of *KRAS* mutations on pancreatic cancer outcome and provide guidance in improving approaches to target *KRAS* in pancreatic cancer.

## Patients and Methods

The MD Anderson Cancer Center Institutional Review Board (IRB) approved the collection of demographics, clinical, and pathological information under IRB protocol 09–0373 and 2023–0091. Informed consent was waived, as per the IRB guidelines for retrospective studies of previously collected clinical and molecular information. The Palantir Foundry software system (Palantir, Denver, CO) was used to query the MD Anderson EHR to identify patients with a confirmed diagnosis of PDAC who underwent somatic tumor tissue mutation testing at MD Anderson from 3/14/1997 to 4/27/2023 for inclusion in the study.

Patient demographics, histopathology, tumor grade, surgical history, and mutational profiles were collected from the MD Anderson EHR and tumor registry data using the Foundry system. Histologic classification and grade were collected from the patients’ pathology reports. Molecular testing was performed at MD Anderson’s molecular diagnostics laboratory, which is College of American Pathologists (CAP) accredited and Clinical Laboratory Improvement Amendments (CLIA) certified. The gene panels used evolved during the study inclusion period, with expanding lists of genes over time. The information on tumor genomic alterations (GAs) was extracted from the available clinical and molecular data. Deidentified information was used for analysis.

For the co-mutation analysis, only patients who were tested with multigene panels were included (n=513). The Oncoplot function within MAFtools was used to visualize the somatic mutation distribution. The function performs pair-wise Fisher’s exact test to uncover mutually exclusive or co-occurring gene sets and an FDR – corrected p<0.1 was considered significant. To better understand the co-mutations patterns with *KRAS* and the rest of the genes, a heatmap was constructed to demonstrate the co-mutation landscape of *KRAS* mutation status, as well as the status of the different *KRAS* alleles, and the rest of the genes (Fig 4B). The percentage of co-occurrence between *KRAS* alleles and pathogenic mutations in the genes listed in the heatmap were determined using in-house R scripts. Fisher’s exact test was used to test for significance in co-occurrence between *KRAS* alleles and pathogenic mutations. Based on the co-mutation patterns observed, we divided patients into 4 molecularly distinct PDAC co-mutation subtypes to visualize and test the relationship between co-mutation pattern and OS.

### Statistical Analysis

Differences in disease stage and tumor grade between patients with different *KRAS* mutations were assessed using Chi-square and Fischer’s exact test. Overall survival (OS, from the time of initial diagnosis) was calculated from the date of initial diagnosis until death or last known contact. OS curves were estimated using the Kaplan-Meier method, and the difference in survival curves was tested using the log-rank test. Univariate Cox proportional hazards models were used to estimate hazard ratios (HRs) and test the associations of *KRAS* mutation status, *KRAS* mutation allele subtypes, and other driver mutations with OS.

In the co-mutation analysis, the somatic interactions function within MAFtools was used to detect mutually exclusive or co-occurring mutation events. Pair-wise Fisher’s exact test was used to uncover mutually exclusive or co-occurring gene sets with Benjamini-Hochberg multiplicity correction, and a false discovery rate (FDR)-corrected p<0.1 was considered significant. The OS curves for the 4 co-mutation subtypes were estimated with the Kaplan-Meier method and compared using the log-rank test.

GraphPad Prism version 9 (GraphPad Software, San Diego, California USA) and Rstudio 2020 (RStudio, PBC. Boston, MA) were used for the statistical analyses and data visualization^73^. All tests were two-sided, and statistical significance was identified by a p-value < 0.05.

### PanCAN’s Know Your Tumor^®^ Program and Dataset

PanCAN, in partnership with Tempus (Tempus Labs Inc., Chicago, IL), offers the Know Your Tumor^®^ (KYT) precision medicine service to patients with pancreatic cancer. KYT data is available through the PanCAN SPARK platform (www.pancan.org/spark). Tempus processes, sequences and conducts group-level bioinformatics analyses on tumor biopsy samples. Data is derived from the Tempus xT NGS panel that covers 648 genes with actionable oncologic mutations. Variants are called from the resulting alignment files using an analysis pipeline that detects SNPs and indels using Freebayes and Pindel^74,75^. A filtered variant file which contains biologically relevant DNA variants, as determined by the Tempus pipeline, were used for all KYT related analyses. Patients with PDAC who had their tumor sequenced by Tempus were included in the analysis. Pathogenic or likely pathogenic mutations were determined by Tempus’ proprietary Knowledge Database which is based on the American College of Medical Genetics and Genomics (ACMG) and Association for Molecular Pathology (AMP) guidelines for variant classification. All mutation data was converted to Mutation Annotation Format (MAF) to enable use of the functions in the Bioconductor R package, MAFtools^76^. The Oncoplot function within MAFtools was used to visualize the somatic mutation distribution across the KYT cohort. The somatic interactions function within MAFtools was used to detect mutually exclusive or co-occurring mutation events. The function performs pair-wise Fisher’s exact test to uncover mutually exclusive or co-occurring gene sets with Benjamini-Hochberg multiplicity correctio and an FDR-corrected p<0.1 was considered significant. The percentage of co-occurrence between *KRAS* alleles and pathogenic mutations in the genes listed in the heatmap in Figure 6 were determined using in-house R scripts. Fisher’s exact test was used to test for significance in co-occurrence between *KRAS* alleles and other pathogenic mutations. Overall survival (OS, from the time of initial diagnosis) was calculated from the date of initial diagnosis until death or last known contact. OS curves by *KRAS* mutation and subtype status were estimated using the Kaplan-Meier method, and the difference in survival curves was tested using the log-rank test.

## Figures and Tables

**Table 1.** MD Anderson Cohort Patient Characteristics

All		803 (100%)
**Age, years- median (range)**		63 (26, 86)
**Race/ethnicity**		
	Asian	56 (7.0%)
	Black or African	55 (6.8%)
	Hispanic or Latino	74 (9.2%)
	White or Caucasian	587 (73.1%)
	Other	31 (3.9%)
**Sex**		
	Female	345 (43.0%)
	Male	458 (57.0%)
**Smoking status**		
	Current Smoker	36 (4.5%)
	Former Smoker	199 (24.8%)
	Never	372 (46.3%)
	Not Available	196 (24.4%)
**Histology grade**		
	Well differentiated	29 (3.6%)
	Moderately differentiated	300 (37.4%)
	Poorly differentiated	321 (40.0%)
	Undifferentiated	4 (0.5%)
	Not Available	149 (18.6%)
**Stage at diagnosis**		
	I	122 (15.2%)
	II	138 (17.2%)
	III	105 (13.1%)
	IV	336 (41.8%)
	Not available	102 (12.7%)
**KRAS (n=703)**		
	Mutant	578 (82%)
	Wildtype	125 (18%)
**TP53 (n=604)**		
	Mutant	418 (69%)
	Wildtype	186 (31%)
**CDKN2A (n=509)**		
	Mutant	102 (20%)
	Wildtype	407 (80%)
**SMAD4 (n=536)**		
	Mutant	68 (13%)
	Wildtype	468 (87%)
**OS, months – median (range)**		19 (0.07, 348)

**Table 2.** PanCan Validation Cohort Patient Characteristics

All		408 (100%)
**Age, years- median (range)**		65(36,88)
**Race/ethnicity**		
	Asian	8 (2.0%)
	Black or African	12 (2.9%)
	Hispanic or Latino	4 (1.0%)
	White or Caucasian	174 (42.6%)
	Other	1 (0.2%)
	Not Available	213 (52.2%)
**Sex**		
	Female	188 (46.1%)
	Male	220 (53.9%)
**Histology grade**		
	Well differentiated	6 (1.5%)
	Moderately differentiated	78 (19.1%)
	Poorly differentiated	46 (11.3%)
	Not Available	278 (68.1%)
**Stage at diagnosis**		
	I	17 (4.2%)
	II	16 (3.9%)
	III	34 (8.3%)
	IV	97 (23.8%)
	Not available	244 (59.8%)
**KRAS (n=408)**		
	Mutant	377 (92.4%)
	Wildtype^	31 (7.6%)
**TP53 (n=408)**		
	Mutant	316 (77.5%)
	Wildtype^	92 (22.5%)
**CDKN2A (n=408)**		
	Mutant	86 (21.1%)
	Wildtype^	322 (78.9%)
**SMAD4 (n=408)**		
	Mutant	97 (23.7%)
	Wildtype^	311 (76.2%)
**OS, months – median (range)**		22 (0.2, 93)

^ Wildtype: Denotes no pathogenic mutations were detected.
